# Epidemiological investigation of *Mycoplasma Synoviae* in native chicken breeds in China

**DOI:** 10.1186/s12917-017-1029-0

**Published:** 2017-04-26

**Authors:** Shi-Kai Sun, Xin Lin, Feng Chen, Ding-Ai Wang, Jun-Peng Lu, Jian-Ping Qin, Ting-Rong Luo

**Affiliations:** 1 State Key Laboratory for Conservation and Utilization of Subtropical Agro-Bioresources, Guangxi University, Nanning, Guangxi 530004 China; 2 Guangdong Enterprise Key Laboratory for Animal Health and Environmental Control, WENS Group Academy, Yunfu, Guangdong 527439 China; 3 College of Animal Science, South China Agricultural University, Guangzhou, Guangdong 510642 China

**Keywords:** *Mycoplasma synoviae*, Outbreak, Chinese native chicken breeds, Epidemiology

## Abstract

**Backgroud:**

*Mycoplasma synoviae* (*M. synoviae*) is widely distributed around the world, and leads to serious economic losses in the world every year. Nevertheless, the incidence and epidemiology of *M. synoviae* infection in China have remained unclear.

**Results:**

In this study we demonstrate that over 9773 broiler chicken flocks in 16 Chinese provinces were affected by *M. synoviae* between 2010 and 2015. Our epidemiological study revealed that *M. synoviae* was widely prevalent in multi-aged Chinese native breeder chickens, and the prevalence of *M. synoviae* in embryos of breeders reached up to 16.29%. In addition, our data showed that chickens aged 14 days or younger carried simultaneously high levels of maternal antibody against *M. synoviae* and high *M. synoviae* infection (10%), and low *M. synoviae* antibody levels in breeders and high proportion of *M. synoviae* infection in embryos could increase the chances of incidence in the offspring. Finally, our results also indicated that 3- to 7-week-old chickens might be most the susceptible to *M. synoviae* and, therefore, might play a key role in the horizontal transmission of *M. synoviae*.

**Conclusion:**

Our findings suggest that *M. synoviae* is widely circulating in Chinese native chickens, accordingly, effective control measures are urgently needed to control the spread.

## Backgroud

*Mycoplasma synoviae* (*M. synoviae*) is an important disease, causing enormous economic losses to the poultry industry worldwide. *M. synoviae* infection in chickens or turkeys results in acute or chronic infectious synovitis or air sacculitis [1].

*M. synoviae* was reported in Australia, South America, Asia, Europe and Africa [2], nevertheless, *M. synoviae* distribution appeared regional in most cases, and large-scale outbreaks rarely occurred. In the past ten years, it seems that *M. synoviae* had taken over the role of *Mycoplasma gallisepticum* in commercial poultry [3]. Apart from air sacculitis and synovitis, eggshell apex abnormalities (EAA) and egg drop syndrome resulting from *M. synoviae* infection have been encountered worldwide [3–6]. Hence, there has been a growing emphasis on understanding the prevalence of *M. synoviae*, especially its subclinical infection [3, 7]. Many reports [8–11] world with a varying degree of incidence in multi-age white-feather chickens or turkeys. To date, there has been no data about *M. synoviae* infection in Chinese native chicken breeds, and the incidence and epidemiology of *M. synoviae* in China are poorly understood.

Between 2010 and 2015, a disease, characteristic of infectious synovitis occurred in native-type chickens in China. The disease resulted in the loss of millions of chickens in Chinese poultry farms. In this study, we conducted systematic epidemiological analysis of this disease.

## Methods

### Sample preparation

#### Identification of pathogen

To determine the causative agent of disease, a total of 18,063 clinical samples (joint fluid swabs and three swabs were collected from three sick chickens at each farm) were collected from infected chickens in the farms (Table 1) of 16 Chinese provinces between 2010 and 2015. In addition, from 2010 to 2013, 1696 serum samples were collected from 53 infected broiler flocks (Table 2). Thirty-two blood samples were collected from each farm, including 16 samples from sick chickens with typical clinical signs and 16 samples from chickens without any clinical symptoms.

**Table 1:** Information of samples for PCR detection and strains isolation

Province	Sampling city	Breed	Age (day)	Joint fluid swabs^a^	Chicken legs^a^	No. of farm	Sampling time
Guangdong	Yunfu, YangJiang, Heyuan, Zhaoqing, Foshan, Qingyuan, Jiangmen	Three-Yellow, Chaohua, Silky, Qingyuan Ma, Wenchang, Tianlushan, Short footed	29–70	5934	61	1978	2010.10–2014.12
Guangxi	Nanning, Guilin, Hezhou, Yulin	Three-Yellow, Chaohua, Silky, Qingyuan Ma, Short footed	31–75	2004	24	668	2010.09–2014.12
Zhejiang	Quzhou, Huzhou, Jiangshan	Ditto	30–65	1233	24	411	2012.03–2014.12
Jiangsu	Zhengjiang, Suzhou, Nanjing, Yancheng, Suqian, Huaian, Nantong, Lianyungang,	Ditto	28–68	1809	14	603	2012.01–2014.12
Hunan	Hengyang, Changsha	Ditto	30–65	1407	16	469	2013.01–2014.12
Hubei	Wuhan, Jingzhou	Three-Yellow, Chaohua, Silky, Qingyuan Ma, Wenchang, Tianlushan, Short footed	42–58	468	6	156	2013.02–2014.12
Anhui	Hefei, Wuhu, Haozhou, Chuzhou	Ditto	33–71	900	4	300	2013.01–2014.12
Jiangxi	Nanchang, Jian	Ditto	30–60	1110	8	370	2013.04–2014.12
Sichuang	Meishan, Deyang, Chengdu	Ditto	32–66	921	12	307	2011.06–2014.12
Yunnan	Kunming, Dali	Ditto	26–71	504	4	168	2012.04–2014.12
Guizhou	Qingzhen, Guiyang	Ditto	31–56	156	5	52	2011.08–2014.12
Chongqing	Chongqing	Ditto	30–64	297	4	99	2012.03–2014.12
Fujian	Putian, Zhangzhou	Three-Yellow, Chaohua, Silky, Qingyuan Ma, Short footed	30–75	723	21	241	2013.02–2014.12
Shangdong	Taian	Three-Yellow, Tianlushan,	30–52	81	3	27	2013.11–2014.12
Hebei	Cangzhou	Three-Yellow, Silky, Qingyuan Ma	33–61	384	4	128	2011.06–2014.12
Hainan	Haikou, Qionghai	Three-Yellow, Wenchang	45–65	132	4	44	2014.03–2014.12
Total				18,063	215	6021	2010–2014

^a^Joint fluid swabs were collected for PCR detection from 2010; ^b^ Chicken legs were collected for strain isolation between 2013 and 2014

**Table 2:** *M. synoviae* antibody of native broilers infected by infectious synovitis in China

Group	Breed	No. of farm^a^	Age (day)	Clinical signs	Type of sample	No. of positive *M. synoviae* antibody	Positive proportion	Sampling time
A1	Tianlu	8	35–60	Arthrocele	Serum	79/103	76.70%	2012.01–2014.12
A2	Tianlu		35–60	NO	Serum	88/131	67.18%	2012.01–2014.12
B1	San Huang	17	30–75	Arthrocele	Serum	208/299	69.57%	2010.10–2014.12
B2	San Huang		30–75	NO	Serum	78/252	30.95%	2010.10–2014.12
C1	Silky	5	31–63	Arthrocele	Serum	59/62	95.16%	2012.06–2014.12
C2	Silky		31–63	NO	Serum	30/80	37.5%	2012.06–2014.12
D1	Hua	9	40–71	Arthrocele	Serum	140/155	90.32%	2012.02–2014.12
D2	Hua		40–71	NO	Serum	81/128	63.28%	2012.02–2014.12
E1	Qingyuan Ma	14	29–62	Arthrocele	Serum	197/240	82.08%	2011.03–2014.12
E2	Qingyuan Ma		29–62	NO	Serum	112/238	47.06%	2011.03–2014.12
Total		53				1072/1696	63.20%	2010.10–2014.12

^a^Thirty-two blood samples were collected from each farm, including 16 samples from sick chickens with typical clinical signs and 16 samples from chickens without any clinical symptoms

#### Prevalence in multi-aged breeder farms

According to the protocol for an epidemic outbreak, a total of 5760 serum samples were collected from 180 breeder flocks in 6 large-scale multi-age native chicken farms in Guangdong, Guangxi, Hunan and Zhejiang, where the disease occurred in 2013, and detected by ELISA. Chickens ranged from 1 to 40 weeks old, 3–4 flocks were examined for each age (week), totaling 180 flocks with 32 blood samples from each flock. In addition, a total of 1332 7- to 9-day-old embryos (Table 3) were collected from farms in Guangxi, Guangdong, Zhejiang and Hunan in 2013. Allantoic fluid was collected from each embryo for detection of *M. synoviae* infection.

**Table 3:** Infection proportion of *M. synoviae* in embryos of 7 breeder farms in 2013

Province	County	No. of farm^a^	No. of positive samples	Positive prevalence	Sampling time
Guangdong	Xinxing, Gaoming	2	92/250	36.80%	March, November
Hunan	Ningxiang	1	4/180	2.22%	March
Guangxi	Yongning	1	73/360	20.27%	March
Zhejiang	Nanxun, Wuxing	3	48/542	8.85%	March, July, August
	Total		217/1332	16.29%	March–November

^a^The 7–9 day-old embryos were collected from 7 breeder farms. Allantoic fluid was collected from each embryo for detection of *M. synoviae* infection by PCR

#### Natural infection in the offspring of native breeders

To determine the natural prevalence of *M. synoviae* in broilers, three batches of 1-day-old Three-Yellow chickens (according to the high incidence of *M. synoviae* infection in Three-Yellow broiler chickens) were purchased from a farm in 2014, with 100 chickens in each batch and a 30-day time interval between each batch. Throat swab samples and serum samples of all chickens were collected at 1, 5, 10, 20, 30, 40, 50, 60, 70, 80, 90 and 100 days old, respectively.

#### Prevalence in different native breeders

To further understand *M. synoviae* infection in different native chicken breeds from a farm in Zhejiang, serum and associated embryos collected from three different native chicken breeds (Three-Yellow, Silky, and Qingyuan Ma chickens) were examined. A total of 128 serum samples were simultaneously collected from 3 different chicken breeds (a total of 8 breeder flocks) on December 11, 2013. A total of 223 7- to 9-day-old embryos corresponding to these breeder flocks were collected on December 20, 2013. Allantoic fluid was collected from each embryo. Subsequently, 12 offspring broiler flocks corresponding to the breeder flocks mentioned above were tracked for the change of *M. synoviae* antibody. A total of 478 serum samples were collected from offspring broiler flocks at 4, 5, 6, 7 weeks old, respectively.

#### ***Samples*** detection

The presence of *M. synoviae* DNA in swab samples and allantoic fluid was determined by PCR. The presence of *M. synoviae* antibody in serum samples was determined by ELISA.

### Isolation and culture of *M. synoviae*

A total of 215 joint fluid swabs were sterile collected from the leg joints of *M. synoviae*-infected chickens from farms in Zhejiang, Hunan, Jiangsu, Anhui, Sichuan, Yunnan, Guizhou, Chongqing, Hubei, Hebei, Jiangxi, Fujian, Shandong, Hainan, Guangdong and Guangxi provinces were collected in 2013–2014 (Table 1). One swab sample was added to a tube containing 2 mL Frey mycoplasma medium (BD Biosciences) with 10% serum and 1% thallium-acetate [2]. This sample was then transferred into a 37 °C incubator (5% CO_2_ atmosphere conditions) to culture for 20–24 h; the sample supernatant was removed when the liquid sample color changed from red to brown. After the 4th or 5th passage, pure culture of isolates was used as template and identified directly by PCR and sequenced.The 4th or 5th passage live cell titer was determined based on 50% color change unit (CCU_50_), as described previously [12, 13].

### DNA extraction, PCR and sequencing

Total DNA was extracted directly from joint swab samples and allantoic fluid using the RNeasy kit (AxyPrep, Union City, USA). The presence of *M. synoviae* DNA in swab samples and allantoic fluid was determined by PCR.The TaKaRa PrimeScript one-step PCR Kit Ver.2 (TaKaRa, Dalian, China) was used for PCR. All PCR reactions were carried out using a PCR machine (Biometra, Goettingen, Germany). Samples were detected by PCR as described by Ramirez et al. [14]. A partial sequence of the *vlhA* gene (1284–1325 bp) of positive samples was amplified by PCR for sequencing as described previously [15]. All sequences from independent PCR products were sequenced 3–4 times in both directions by Sangon Biotech (Shanghai) Co., Ltd.

### Sequence analysis

After sequencing PCR fragments, nucleotide sequence editing was accomplished using DNAStar (Madison, WI). Multiple-sequence alignments were generated with ClustalX 2.0, and nucleotide sequence homologies were obtained using the Clustal W method of DNAStar. All sequencing data were assembled using DNAStar (Madison, WI). The sequence of each different strain was compared to reference isolates in GenBank.

### Serological study

Serum samples were tested for the presence of *M. synoviae* antibodies using the *M. synoviae* Antibody test kit (IDEXX, Liebefeld-Bern, Switzerland) according to the manufacturer’s instructions. Based on the optical density (*OD*) values at 405 nm, sample to positive (S/P) ratios were calculated, and the averaged S/P ratio was used to evaluate the antibody level in each group. Serum samples with S/P ratios greater than 0.2 (titers greater than 1076) were considered to contain anti-*M. synoviae* antibodies, and a ratio of 0.2 or lower (titers less than or equal to 1076) were considered negative, based on the manufacturer’s recommendations.

### In vivo experiments

WVU1853 (*M. synoviae* standard strain, bought from China Veterinary Drug Supervision), 14 randomly chosen *M. synoviae* isolates and 160 20-day-old SPF chickens (specific pathogen free chickens, Guangdong Dahuanong Co., LTD) were used for the pathogenicity experiments. 160 chickens were divided into 16 groups, including 15 challenged groups (10 birds/group) and one mock/control group (10 birds). All chickens were kept in SPF chicken isolators (glove box) through the whole experiment. After adaption for three days in the isolator, the chickens were inoculated with fresh cultures of 14 selected isolates and WVU1853 by intramuscular injection with a dose of 10^7^ CCU_50_. Ten chickens served as mock controls and were mock-injected with 0.5 mL culture medium in the same manner. The chickens were monitored daily while clinical symptoms and morbidity were recorded. All chickens were necropsied on day post-inoculation.

### Statistical analysis

The data obtained were analyzed by one-way analysis of variance (ANOVA), and the differences between means were compared by Duncan’s multiple range test (DMRT) using SPSS 17 (SPSS Inc., Chicago, IL, USA). *M. synoviae* antibody titer and *M. synoviae* antigen-carrying rate were analyzed by Student’s t-test. *P* < 0.05 was considered to be statistically significant.

## Results

### Identification of infectious synovitis caused by *M. synoviae* infection

**Fig. 1:**
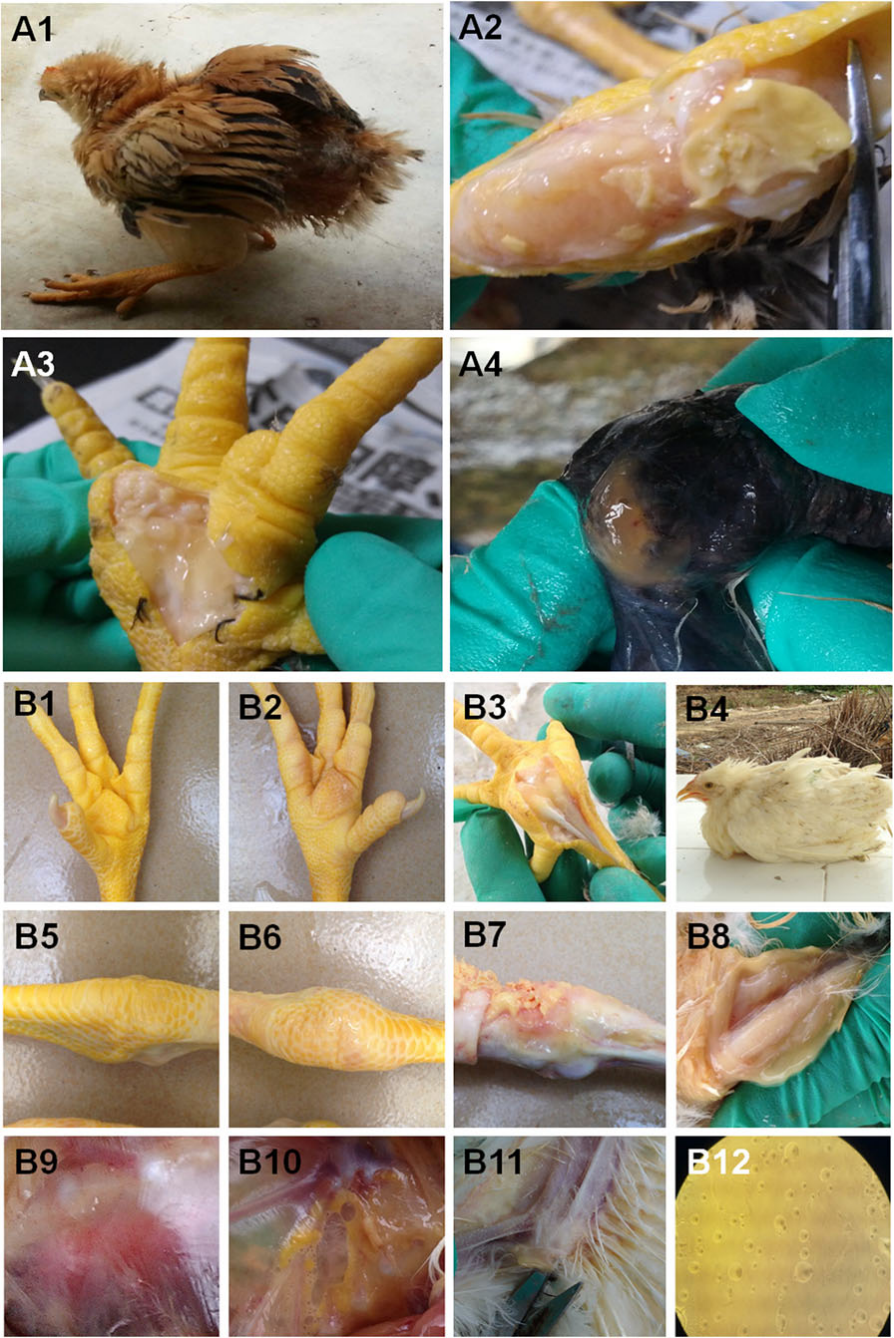
Pathological studies of infected chickens and inoculated chickens.The infected chickens, which showed clinical symptoms including movement disorders, erect feathers and stunted growth (**a1**), were killed, and anatomical investigations revealed the presence of thick, clear to milky exudate in enlarged joints (**a2**, **a4**) and foot pads (**a3**). Similar to the clinically infected chickens, the inoculated chickens exhibited symptoms such as movement disorders, erect feathers and stunted growth (**b4**). The anatomical investigations showed that thick, clear to milky exudate was present in enlarged foot pads (**b2**, **b3**), joints (**b6**, **b7**), keels (**b8**) and wings (**b11**). In addition, air sacculitis (**b10**) was observed in infected chickens too. Mock-injected chickens’ foot pads (**b1**), joints (**b5**) and air sacs (**b9**) were compared with those of the infection chickens. Colonies of *M. synoviae* were grown in solid medium (**b12**)

**Fig. 2:**
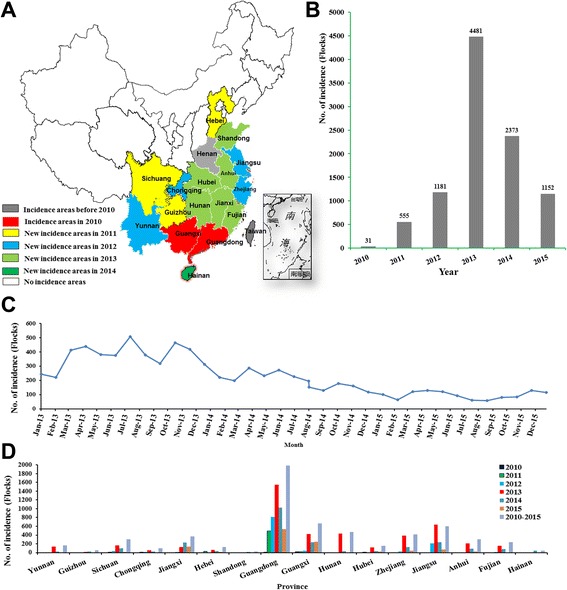
The prevalence of *M. synoviae* infection in China between 2010 and 2015.**a** Incidence in various areas of China from 2010 to 2015; **b**Total cases of infectious synovitis that appeared in China from 2010 to 2015; **c** Monthly case occurrence during 2013 and 2015 in China. **d** Overall cases that appeared in different provinces with reported incidents. (We acknowledge Tang HB for providing the original map of China. The original map was published in the paper by Tang HB et al.: Re-emergence of rabies in the Guangxi province of Southern China. PLoS Negl Trop Dis 2014.8(10):e3114)

**Fig. 3:**
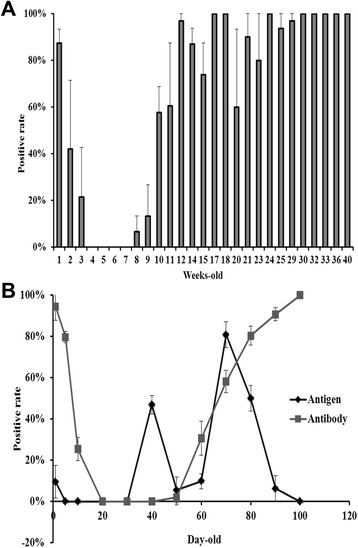
Prevalence of *M. synoviae* infection in multi-aged Chinese native chicken breeds and breeder offspring. **a** *M. synoviae* antibody levels of multi-aged ages breeder farms in Hunan, Guangdong, Guangxi and Zhejiang provinces in contemporaneity, 2013; **b** Changes of *M. synoviae* infection proportion and *M. synoviae* antibody levels in monitored flocks (Three-Yellow fowl) from 1 to 100 days-old in Yunfu, Guangdong, 2013. *P* < 0.05

**Fig. 4:**
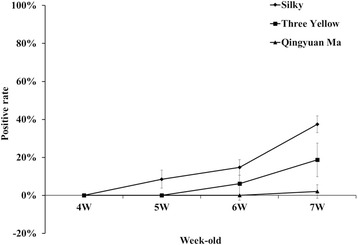
Changes in *M. synoviae* antibody levels in several clinical native breed chickens.The 3 breeds of broiler (a total of 12 flocks), including Three-Yellow-, Silky-, and Qingyuan Ma-chicken, were tracking for change of *M. synoviae* antibody at 4, 5, 6, 7 weeks old in the production process, respectively. Positive *M. synoviae* antibody was first observed in the Silky chicken flocks (at 5 weeks-old), but last in the Qingyuan Ma-chicken flocks (at 7 weeks-old). *P* < 0.05

In 2010, a disease with characteristic infectious synovitis appeared in chickens in the Guangdong and Guangxi provinces of China (Fig. 1a1). Anatomical examination revealed that visible yellowish purulent exudate or a yellow cheese-like substance accumulated in the joint and wing cavities of sick chickens (Figs. 1a, 2, 3, 4). This disease had a mass rate of 10–100% and a group morbidity rate reaching 1–20%, with an average of 5–8%. This disease was observed in several Chinese native chicken breeds, including Three-Yellow, Silky, Qingyuan Ma, Wenchang, Tianlushan, Chaohua and Short-footed chickens.

To determine the causative agent of this disease, we first investigated several pathogens that had previously been reported to associate with infectious synovitis in broiler chickens by pathogenic detection, including *Salmonella* [16]*, Reovirus* [17, 18]*, Streptococcus* [19, 20]*, Staphylococcus* [21]*,* and *M. synoviae* [2]*.*The results indicated that no of these pathogens could be detected (data not shown). By contrast, we found that only *M. synoviae* DNA was detected in most of the infected chickens (17,396/18063, 96.31%). Serum antibody analysis also showed the presence of *M. synoviae* antibody in 69.57–95.16% of the infected chickens, compared to 30.95–67.18% in clinically healthy chickens of the same flocks (Table 2). The highest prevalence of *M. synoviae* antibody was observed in silky chickens and the lowest in Three-Yellow chickens (Table 2). 110 *M. synoviae* strains were successfully isolated from *M. synoviae*-infected chickens (Fig. 1b12) and sequenced (Genbank accession number: KU572280-KU572389). Subsequently, typical clinical symptoms were reproduced using part of isolates (Fig. 1b4). Similar to the clinical cases, anatomical examination of experimentally infected chickens revealed that a pale yellow or yellow purulent exudate, cheese-like substance was deposited in the feet, leg joints, wings, and chest (Figs. 1b2, 3, 6, 7, 8, 11), and a proportion of the chickens also exhibited air sacculitis (Fig. 1b10). The experimental *M. synoviae* strains were successfully recovered from the experimentally infected chickens, and the diagnosis of *M. synoviae* infection was confirmed by *M. synoviae*-specific PCR and gene sequencing in subsequent experiments (data not shown).

The results revealed that the outbreak of infectious synovitis that emerged in China from 2010 to 2015 was caused by *M. synoviae* infection.

### Prevalence of *M. synovia*e infection between 2010 and 2015

In 2010, the disease initially appeared in the Guangdong and Guangxi provinces of China. The disease spread to larger areas over time, as evidenced by the observation of cases in new provinces since 2011, including Fujian, Zhejiang, Hunan, Jiangsu, Anhui, Sichuan, Yunnan, Guizhou, Chongqing, Hubei, Hebei, Jiangxi, Fujian, Shandong, and Hainan provinces (Fig. 2a). Our epidemiological study showed a dramatic increase in the number of cases from 2010 to 2013 in China, and a slight decrease in 2014 and 2015 (Fig. 2b). The increase may have been associated with a wide range of chicken breeds and a large-scale of cultivation. The disease had no obvious seasonal or breed specificity but did show a slight increase in prevalence at the turn of seasons, i.e. March, April, June, July, September and October (Fig. 2c). The most severe outbreak of the disease occurred in 2013, with the monthly average number of infected flocks reaching approximately 400 (Fig. 2d). It is worth mentioning that Guangdong province, where the incidence was the most serious, had 4417 infected flocks from 2010 to 2015; in 2013 alone, a total of 1547 flocks were infected (Fig. 2d).

Though strong comprehensive measures for prevention and control (including the use of antibiotics and strict implementation of sanitation and disinfection) were carried out after the outbreak of *M. synoviae*, there was still relatively high morbidity in China every year (Fig. 2). Hence, further outbreaks of infectious synovitis have emerged in several native-type chickens in China.

### *M. synoviae* infection prevalence in multi-aged breeder farms

The results indicated that chickens within a week old normally carried high levels of maternal *M. synoviae* antibody, which declined gradually over the next two weeks. *M. synoviae* antibody levels in chickens remained negative between 4 to 8 weeks old. The average positive antibody rates of 8-, 9-, 10-, 11-, 12-, 13-, 14-, 15-, 20-, 21-, 23-, 25-, and 29-week-old chickens were 7%, 13%, 58%, 60%, 97%, 87%, 74%, 60%, 90%, 80%, 94%, and 97%, respectively. The average positive antibody rates of 17-, 18-, 24-, 30-, 32-, 33-, 36-, 40-week-old chickens all reached 100%. *M. synoviae* antibody levels in 13- to 25-week-old chickens showed large antibody levels, especially between 20 and 25 weeks old. Multiple variant *M. synoviae* antibody levels were observed among flocks of different ages in this period of time (Fig. 3a).

Subsequently, 1332 embryos from these 6 farms were collected for *M. synoviae* DNA detection. The results showed that the proportion of embryos from Guangdong, Guangxi, Hunan, Zhejiang provinces exhibiting *M. synoviae* infection was 36.8%, 20.27%, 2.22% and 8.85%, respectively (Table 3). In addition, the rate of occurrence of eggs with rough head in breeder flocks with lower antibody levels was higher, and the prevalence of *M. synoviae* infection in rough head eggs was significantly higher than that of normal eggs.

These results indicated that Chinese native-breed chickens could carry high levels of *M. synoviae* maternal antibody before the age of 14 days, and there was a widespread *M. synoviae* infection and varying degrees of *M. synoviae* vertical transmission in multi-age breeder farms.

### Natural *M. synoviae* infection in the offspring of native breeders

The results showed that the change in *M. synoviae* antibody level in 1- to 20-day-old offspring was consistent with what was observed in the breeder flock described above. The average positive *M. synoviae* antibody rate, which was over 95% for 1-day-old chickens, gradually turned negative before the offspring were 20 days old. The chickens remained negative for the *M. synoviae* antibody between 20 and 50 days old. Then, corresponding to the data from breeders, the average positive antibody rate gradually increased from 0% to 100% in chickens 50 to 100 days old. These results revealed that *M. synoviae* antibody levels exhibited a gradual, age-dependent increase in chicken offspring (Fig. 3b).

At the same time, the results of *M. synoviae* DNA detection showed that the prevalence of infection in chickens also increased quickly as the offspring aged. There were three time points of peak discharge for excreting bacteria: 1 day old (approximately 10%), 40 days old (approximately 50%), and 70 days old (approximately 90%) (Fig. 3b). Then, the chicken stopped excreting bacteria rapidly. Our study confirmed that chickens can simultaneously carry *M. synoviae* maternal antibody and *M. synoviae*, and *M. synoviae* showed a strong capacity for horizontal transmission in Chinese native chicken breeds.

Furthermore, the prevalence of *M. synoviae* infection in naturally infected chickens showed periodic, age-related changes. Three- to seven-week-old chickens might be most susceptible to *M. synoviae* infection and, therefore, might play a key role in the horizontal transmission of *M. synoviae*.

### *M. synoviae* infection of different native chickens on a farm

The results showed that the majority of flocks exhibited high *M. synoviae* antibody levels, and the average positive rate in seized chickens was 73.33–100%. In 30-week-old chickens, the average positive antibody rate could reach 100%, and the average titer could reach 9265 or higher. Flocks of 1005 (Silky chicken) and 1002 (Three-Yellow chicken) had the lowest antibody levels out of all 8 flocks, and the mean titer in these flocks was 2770 and 5648, respectively. Qingyuan Ma chickens had the highest average antibody levels among the three varieties of breeders, while Silky chickens had the lowest (Table 4).

**Table 4:** Survey of *M. synoviae* infection in a farm of Zhejiang in December 2013

Flock	Week-old	Breed	Antibody of serum samples from breeder flock^b^				Infection proportion of *M. synoviae* in hatching embryos^c^		
			Positive rate	AMn^a^	CV	No. of sample	No. of positive	Infection proportion	Infection proportion of breed
1001	24 W	San Huang	100%	5648	40	16	0/27	0.00%	4.63%
1002	29 W	San Huang	100%	8634	47.6	16	2/27	7.41%	
1003	33 W	San Huang	100%	9265	48.2	16	1/24	4.20%	
1004	39 W	San Huang	100%	10,944	40.8	16	2/30	6.67%	
1005	30 W	Silky	73.33%	2770	83.3	16	3/29	10.34%	9.25%
1006	54 W	Silky	100%	16,693	39.7	16	2/25	8.00%	
1007	35 W	Qingyuan Ma	100%	11,545	34.4	16	5/32	15.62%	9.83%
1008	47 W	Qingyuan Ma	100%	11,320	34.9	16	1/29	3.45%	

^a^ Arithmetic mean; ^b^Serum samples were collected from the breeder flocks (1001–1008) on December 11, 2013 and detected by ELISA; ^c^7–9 days-old embryos corresponding to the breeder flocks (1001–1008) were collected on December 20, 2013. Allantoic fluid was collected from embryos for *M. synoviae* antigen detection by PCR on December 20, 2013

The proportion of *M. synoviae* infection in embryos of flocks 1001, 1002, 1003, 1004, 1005, 1006, 1007, and 1008 was 4.2%, 0.00%, 6.67%, 7.41%, 10.34%, 8%, 3.45%, and 15.62%, respectively (Table 4). The prevalence of *M. synoviae* infection in Three-Yellow chickens, Qingyuan Ma chickens and Silky chickens was 3.78%, 9.83%, and 9.25%, respectively (Table 4). The prevalence of *M. synoviae* infection in embryos across all 8 breeder flocks was 7.34%, which was substantially similar to the incidence in broiler chickens (5–8%), which were the offspring of the 8 breeder flocks in Zhejiang Province.

The progeny broilers originating from these three breeds were tracked for analysis of *M. synoviae* antibody within four weeks (in 4- to 7-week-old chickens). It was found that Silky broiler chickens were positive for the *M. synoviae* antibody earlier than the other two breeds. In Silky broilers were positive for the *M. synoviae* antibody at five weeks old, while the other two breeds were positive at six weeks old. The prevalence of the antibody in Three-Yellow chickens, Qingyuan Ma chickens and Silky chickens was 5%, 20% and 40% at seven weeks old, respectively (Fig. 4).

These results indicated that low *M. synoviae* antibody levels in breeders and high *M. synoviae* antigen positive rate in embryos could increase the chance of incidence in the offspring. Thus, *M. synoviae* maternal antibody level and *M. synoviae* infection in embryos might affect the incidence of infection in broiler chickens under threat of *M. synoviae* infection.

## Discussion

In the 1950s, *M. synoviae* infection was first reported in America [22, 23], after that, researchers came to realize its importance in the poultry industry [1, 2, 24, 25]. Prior to this study, there were some single, sporadic reports about white feather chickens affected by *M. synoviae* infection in China [26], but there was a complete lack of data about *M. synoviae* infection in Chinese native chicken breeds. This study is the first to confirm the presence of large epidemics of *M. synoviae* infection in multi-aged farms of native Chinese chicken breeds.

We revealed that chickens could carry the *M. synoviae* maternal antibody and *M. synoviae* simultaneously before 3 weeks of age, and antibody levels of breeders affected the timing of antibody production and clinical incidence in broiler chickens. The incubation period of *M. synoviae* infection by horizontal transmission was usually 11–21 days but was shorter by vertical transmission (up to only 6 days) [2, 27]. Based on these results, *M. synoviae* antibody and the typical clinical signs of *M. synoviae* infection should be observed in chickens due to vertical transmission of *M. synoviae* at approximately 20 days of age. In fact, although there was vertical transmission, the presence of *M. synoviae* antibodies in chickens generally could not be detected until 50 days or even later, and the earliest onset of infection in chickens was also not earlier than 35 days in the clinic. Otherwise, although the proportion of embryos with *M. synoviae* infection could exceed 36%, there was no correspondingly high clinical incidence in broiler chickens of Chinese native breeds. It might suggest that, in addition to the potential of chickens to produce anti-*M. synoviae* antibodies [28] and virulence differences between *M. synoviae* isolates [29–31], maternal antibodies against *M. synoviae* could inhibit *M. synoviae* activity in vivo at early ages and the developmental window when chickens were negative for the *M. synoviae* antibody - might play a key role in *M. synoviae* reproduction in hosts. Therefore, improving maternal antibody levels in developing chickens and effectively controlling *M. synoviae* activity during the developmental window when chickens are negative for the *M. synoviae* antibody might an efficient strategy to prevent *M. synoviae* infection.

Many immunosuppressive pathogens can interact with *M. synoviae* and lead to serious disease in hosts [1, 32]. Co-infection of chickens with *M. synoviae* subclinical infection with avian influenza virus (AIV) H9 [11], infectious bronchitis virus (IBV) [4, 33, 34], Newcastle disease virus (NDV) [34, 35] or infectious bursal disease virus (IBDV) [32] caused more severe respiratory or systemic disease than infection with any single above-mentioned pathogen. Normally, chickens in China usually suffer from the threat of the above-mentioned situation, but the prevalence of subclinical infection in commercial broilers has not been reported. Consequently, the actual economic loss associated with *M. synoviae* infection might be more serious and larger than it is reported in the press. In this case, we need to attach more importance to *M. synoviae* prevention and control strategies in China.

## Conclusions

In this study, we demonstrated that multi-aged breeder farms are widely infected with *M. synoviae* and that *M. synoviae* is circulating in Chinese native-type chickens. Chinese native-type breeders of 14 days old or younger simultaneously carried high levels of maternal antibodies against *M. synoviae*. In addition, vertical transmission of *M. synoviae* was observed, and prevalence of *M. synoviae* infection in embryos of breeders reached as high as 16.29%. Finally, our study suggested that 3- to 7-week-old chickens might be the most susceptible to *M. synoviae* infection and may therefore play a key role in the horizontal transmission of *M. synoviae*.

## Abbreviations

ELISA: Enzyme-linked immunosorbent assay
PCR: Polymerase chain reaction
SPF: Specific pathogen free
CCU_50_: 50% color change unit
SPSS: Statistic package for social science
DNA: Deoxyribose nucleic acid
